# Molecular Phylogenesis and Spatiotemporal Spread of SARS-CoV-2 in Southeast Asia

**DOI:** 10.3389/fpubh.2021.685315

**Published:** 2021-07-30

**Authors:** Mingjian Zhu, Jian Shen, Qianli Zeng, Joanna Weihui Tan, Jirapat Kleepbua, Ian Chew, Jia Xian Law, Sien Ping Chew, Anita Tangathajinda, Natthjija Latthitham, Lanjuan Li

**Affiliations:** ^1^State Key Laboratory for Diagnosis and Treatment of Infectious Diseases, National Clinical Research Center for Infectious Diseases, Collaborative Innovation Center for Diagnosis and Treatment of Infectious Diseases, The First Affiliated Hospital, Zhejiang University School of Medicine, Hangzhou, China; ^2^Shanghai Institute of Biological Products, Shanghai, China; ^3^Faculty of Arts and Social Sciences, National University of Singapore, Singapore, Singapore; ^4^Thammasat University Hospital, Pathum Thani, Thailand; ^5^Zhejiang University School of Medicine, Hangzhou, China; ^6^Tuanku Ja'afar Hospital, Seremban, Malaysia; ^7^Shanghai Jiao Tong University School of Medicine, Shanghai, China

**Keywords:** SARS-CoV-2, phylogenetic tree, phylogeography, phylodynamics, effective population size (Ne), Bayesian inference, Southeast Asia

## Abstract

**Background:** The ongoing coronavirus disease 2019 (COVID-19) pandemic has posed an unprecedented challenge to public health in Southeast Asia, a tropical region with limited resources. This study aimed to investigate the evolutionary dynamics and spatiotemporal patterns of severe acute respiratory syndrome coronavirus 2 (SARS-CoV-2) in the region.

**Materials and Methods:** A total of 1491 complete SARS-CoV-2 genome sequences from 10 Southeast Asian countries were downloaded from the Global Initiative on Sharing Avian Influenza Data (GISAID) database on November 17, 2020. The evolutionary relationships were assessed using maximum likelihood (ML) and time-scaled Bayesian phylogenetic analyses, and the phylogenetic clustering was tested using principal component analysis (PCA). The spatial patterns of SARS-CoV-2 spread within Southeast Asia were inferred using the Bayesian stochastic search variable selection (BSSVS) model. The effective population size (Ne) trajectory was inferred using the Bayesian Skygrid model.

**Results:** Four major clades (including one potentially endemic) were identified based on the maximum clade credibility (MCC) tree. Similar clustering was yielded by PCA; the first three PCs explained 46.9% of the total genomic variations among the samples. The time to the most recent common ancestor (tMRCA) and the evolutionary rate of SARS-CoV-2 circulating in Southeast Asia were estimated to be November 28, 2019 (September 7, 2019 to January 4, 2020) and 1.446 × 10^−3^ (1.292 × 10^−3^ to 1.613 × 10^−3^) substitutions per site per year, respectively. Singapore and Thailand were the two most probable root positions, with posterior probabilities of 0.549 and 0.413, respectively. There were high-support transmission links (Bayes factors exceeding 1,000) in Singapore, Malaysia, and Indonesia; Malaysia involved the highest number (7) of inferred transmission links within the region. A twice-accelerated viral population expansion, followed by a temporary setback, was inferred during the early stages of the pandemic in Southeast Asia.

**Conclusions:** With available genomic data, we illustrate the phylogeography and phylodynamics of SARS-CoV-2 circulating in Southeast Asia. Continuous genomic surveillance and enhanced strategic collaboration should be listed as priorities to curb the pandemic, especially for regional communities dominated by developing countries.

## Introduction

Coronavirus disease 2019 (COVID-19) is an infectious disease, first reported in Wuhan, China, in late December 2019 (1, 2). COVID-19 is caused by a positive-sense, single-stranded RNA (+ssRNA) virus of zoonotic origin (3, 4). Given the close genetic similarity, it was classified as a new member of the genus Betacoronavirus and named severe acute respiratory syndrome coronavirus 2 (SARS-CoV-2) (5). SARS-CoV-2 has a genome of approximately 30 kb, encoding four structural proteins, namely spike (S), membrane (M), envelope (E), and nucleocapsid (N) proteins, as well as several non-structural proteins (6). The S protein on the surface contains the receptor-binding domain (RBD), which is critical for mediating cell entry and is relatively prone to mutations (7).

As a global public health threat, the COVID-19 outbreak was declared a pandemic by the WHO on March 11, 2020 (8). During the first 8 months of 2020, more than 25 million cases and 800,000 deaths were reported worldwide (9). The WHO has reported that 90% of all countries experienced disruptions to essential health services, with low- and middle-income countries (LMICs) facing the greatest difficulties (10). The contribution of Southeast Asia, which is dominated by underdeveloped economies, to the global total of COVID-19 cases is nearly 2% at the time of writing since the first infection within the region was detected in Thailand on January 13, 2020 (9, 11). Unlike in other regions, the epidemic situation in Southeast Asia is unevenly distributed and is subject to further uncertainties (11, 12).

Genomic epidemiology, particularly phylodynamic analysis, is crucial for inferring evolutionary history and revealing epidemic patterns (13–15). Bayesian inference, incorporating prior information, has been applied to the phylodynamics of viral infectious diseases, including COVID-19 (16–18). During the ongoing COVID-19 pandemic, highly informative genome sequence data were generated and publicly shared through online platforms, such as the Global Initiative on Sharing Avian Influenza Data (GISAID). Genomic data revealed a continuous evolution of SARS-CoV-2 in almost all genomic regions, potentially in a country-specific manner (17). Although several COVID-19 genomic epidemiological studies have been conducted, most have focused on North America, Western Europe, and East Asia or individual countries (19–21). A large-scale phylogeographic study reconstructed the worldwide spread of the main SARS-CoV-2 clades; however, it did not provide details on the spread of the virus within each continent (especially within Asia, the largest continent) (22). Yap et al. conducted a study on Southeast Asia; they used phylogenetic and mutational analysis to identify three central variants with possible ongoing adaptation among 142 genomes sampled before mid-April 2020 (23). Islam et al. demonstrated the emergence of European and North American mutant variants of SARS-CoV-2 in South-East Asia and constructed a transmission map using Nextstrain with 329 sequences (24). Limited by the amount of genomic data available, the existing research findings should be considered an early snapshot of the evolution and dissemination of the SARS-CoV-2 strains circulating in the region. Moreover, the geographical scope of both studies covers only a few Southeast Asian countries. Thus, an updated and complete picture of SARS-CoV-2 phylodynamics within the region is needed.

In this study, Bayesian methods were applied to bridge current knowledge gaps by inferring the time-scaled phylogeny of SARS-CoV-2 strains and the spread patterns of this pandemic in Southeast Asia, a region relatively ignored by researchers. The results of this study provide valuable insight into the current pandemic and have the potential to inform future epidemic interventions at a regional level for Southeast Asia and beyond.

## Materials and Methods

A workflow schema was created to describe the key implementation steps and logic of this study (Supplementary Figure 1).

### Sequence Data Acquisition and Filtering

SARS-CoV-2 genome sequences were downloaded from the GISAID database (https://www.gisaid.org/) on November 17, 2020. The following inclusion and exclusion criteria were applied to select samples for analysis: (1) they should originate from Southeast Asia (member states of the Association of Southeast Asian Nations and Timor-Leste); (2) only complete viral genomes from infected individuals (more than 29 kb in length) were included; (3) high-quality sequencing and assembly (the proportion of undetermined nucleotide bases <5%) was required; (4) sequences with unknown or incomplete sampling dates were excluded; (5) sequences with Vero cell labels were excluded. After filtering was done, the final dataset comprised 1,491 genomes of SARS-CoV-2 strains from 10 Southeast Asian countries (except Laos). These samples were obtained between January 5, 2020 and August 31, 2020. The distributions of the sequence samples included for analysis are summarized in Supplementary Table 1. In addition, the accession numbers and the XML file are listed as Supplementary Files 1,2, respectively.

### Maximum-Likelihood (ML) Phylogenetic and Temporal Signal Analyses

Sequencing data were aligned using MAFFT v7.467 (Osaka University, Suita, Osaka, Japan) (25). An ML phylogenetic tree was constructed using the IQ-TREE v.2.0.6 software (Free Software Foundation, Boston, Massachusetts, USA) (26). The best-fit nucleotide substitution model (general time reversible with four gamma categories, GTR + G4) was selected according to the Bayesian information criterion (BIC) using the jModelTest v2.1.7 tool (Free Software Foundation, Boston, Massachusetts, USA) (27). A regression of root-to-tip genetic distance against sampling time was performed to evaluate the temporal signal in the dataset using the TempEst v1.5.3 tool (28). The estimated genetic distance was computed based on the maximum composite likelihood metric.

### Time-Scaled Phylogenetic Analysis and Principal Component Analysis (PCA)

Time-scaled phylogenies for whole genomes were analyzed through Bayesian inference combined with Markov chain Monte Carlo (MCMC) methods using the BEAST v1.10.4 tool (29, 30). Bayesian phylogenetic analyses were performed based on the uncorrelated lognormal (UCLN) relaxed clock and three independent MCMC procedures. MCMC chains were run for 120 million steps, sampling every 300 steps from the posterior distribution. The convergence of all parameters was assessed by calculating effective sample sizes, setting their threshold at 200 using the Tracer v1.7.1 tool (30). After discarding the first 50 million steps as burn-in, 700,000 trees were combined using the LogCombiner v1.10.4 tool. The final tree was summarized as maximum clade credibility (MCC) tree with the TreeAnnotator v1.10.4 tool (both tools were used through the BEAST package). Node ages were annotated using median values. Geographic locations of the internal states on the tree were not observed and were treated as hidden states, which are associated with uncertainties. Branches were color-coded based on the locations of sampling for tips.

To assess the reliability of the major clades generated from the time-scaled phylogenetic tree, we performed PCA. Genome-wide variation data were extracted by the global pairwise sequence alignment of 1491 Southeast Asian SARS-CoV-2 genomes against the NC_045512 reference genome (downloaded from NCBI http://www.ncbi.nlm.nih.gov/) using EMBOSS Stretcher v.6.6.0 (European Bioinformatics Institute, Hinxton, Cambridgeshire, England) (31). PCA was performed using the PLINK v1.9 tool (32, 33), and the first three principal components (PCs) were plotted using the SPSS statistical software v26.0 (IBM Corp., Armonk, New York, USA).

### Molecular Phylogeography and Demographic History Analyses

The two-way asymmetric Bayesian stochastic search variable selection (BSSVS) model was used to reconstruct the molecular phylogeography of SARS-CoV-2 within Southeast Asia. The Bayes factor (BF) was used to measure the likelihood of an epidemiological association between two sequence source countries (16). A BF value exceeding 10 (roughly similar to *P* < 0.01) indicates that the epidemiological association is strongly supported by the examined data, whereas a value less than 3 (roughly similar to *P* > 0.05) suggests that the epidemiological association is not statistically significant (16, 34, 35). Regarding demographic history, changes in effective population size (Ne) over time were inferred based on the coalescent theory using the Bayesian Skygrid approach. Both analyses were conducted using the BEAST v1.10.4 software.

To visually display the spatiotemporal spread of the pandemic in Southeast Asia during the study, a dynamic webpage was created using the Apache ECharts v4.1.0 tool (Apache Software Foundation, Forest Hill, Maryland, USA) (36).

## Results

### ML Phylogeny and Temporal Structure

**Figure 1:**
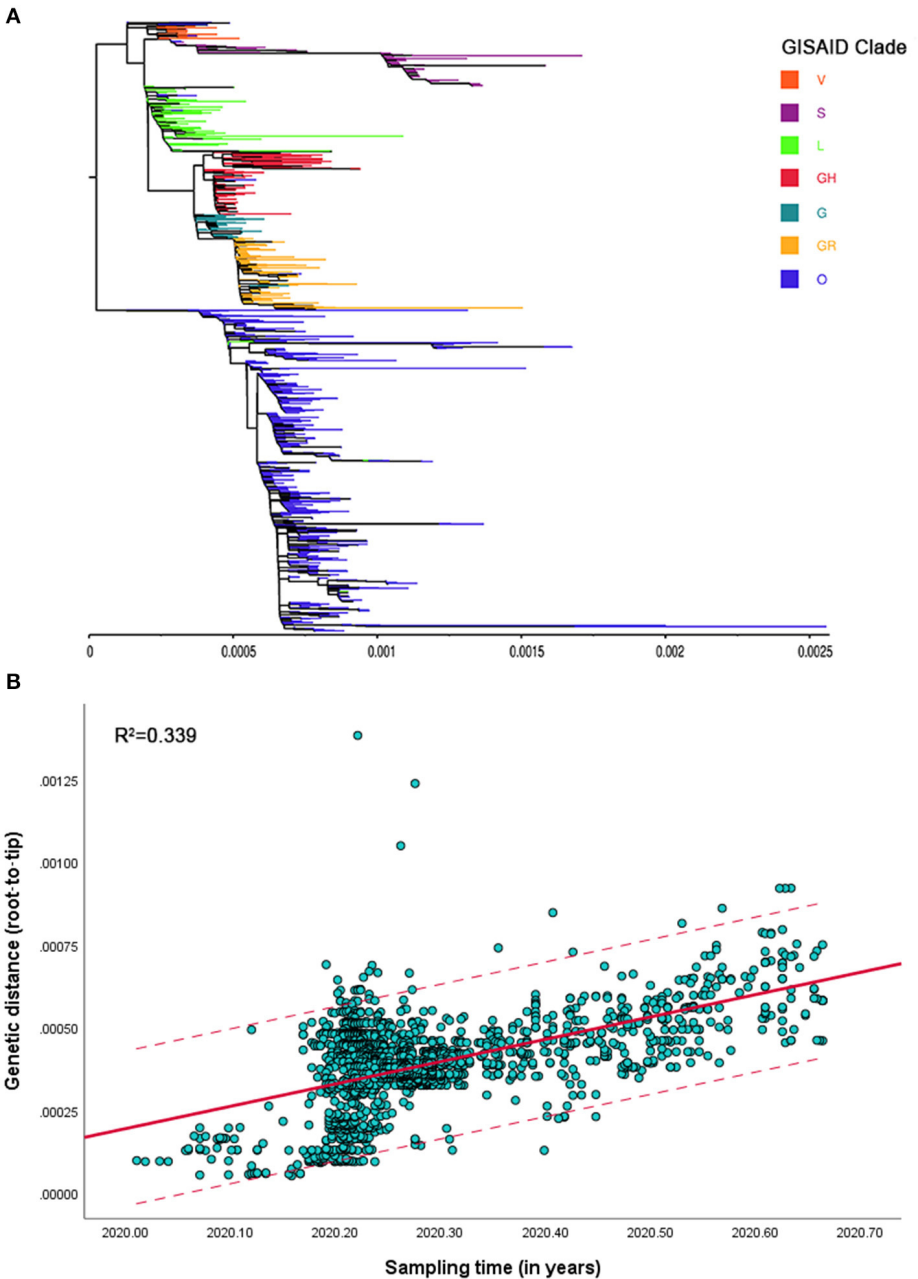
**(A)** Maximum-likelihood (ML) phylogenetic tree of severe acute respiratory syndrome coronavirus 2 (SARS-CoV-2) strains isolated from Southeast Asia using complete genomes. **(B)** Root-to-tip regression based on the ML phylogenetic tree; the solid red line and dotted lines represent the regression line and upper or lower limits of 95% confidence interval (CI), respectively.

The ML phylogenetic tree based on complete genomes showed the molecular evolution of SARS-CoV-2 isolates from Southeast Asia (Figure 1A). Using root-to-tip regression analysis, a positive correlation between genetic divergence and sampling time was found; the retrieved R^2^ value of 0.339 (F = 764.817; *P* < 0.01) suggested the presence of a temporal signal in the dataset (Figure 1B).

### Time-Scaled Phylogenetic Reconstruction of SARS-CoV-2 in Southeast Asia

**Figure 2:**
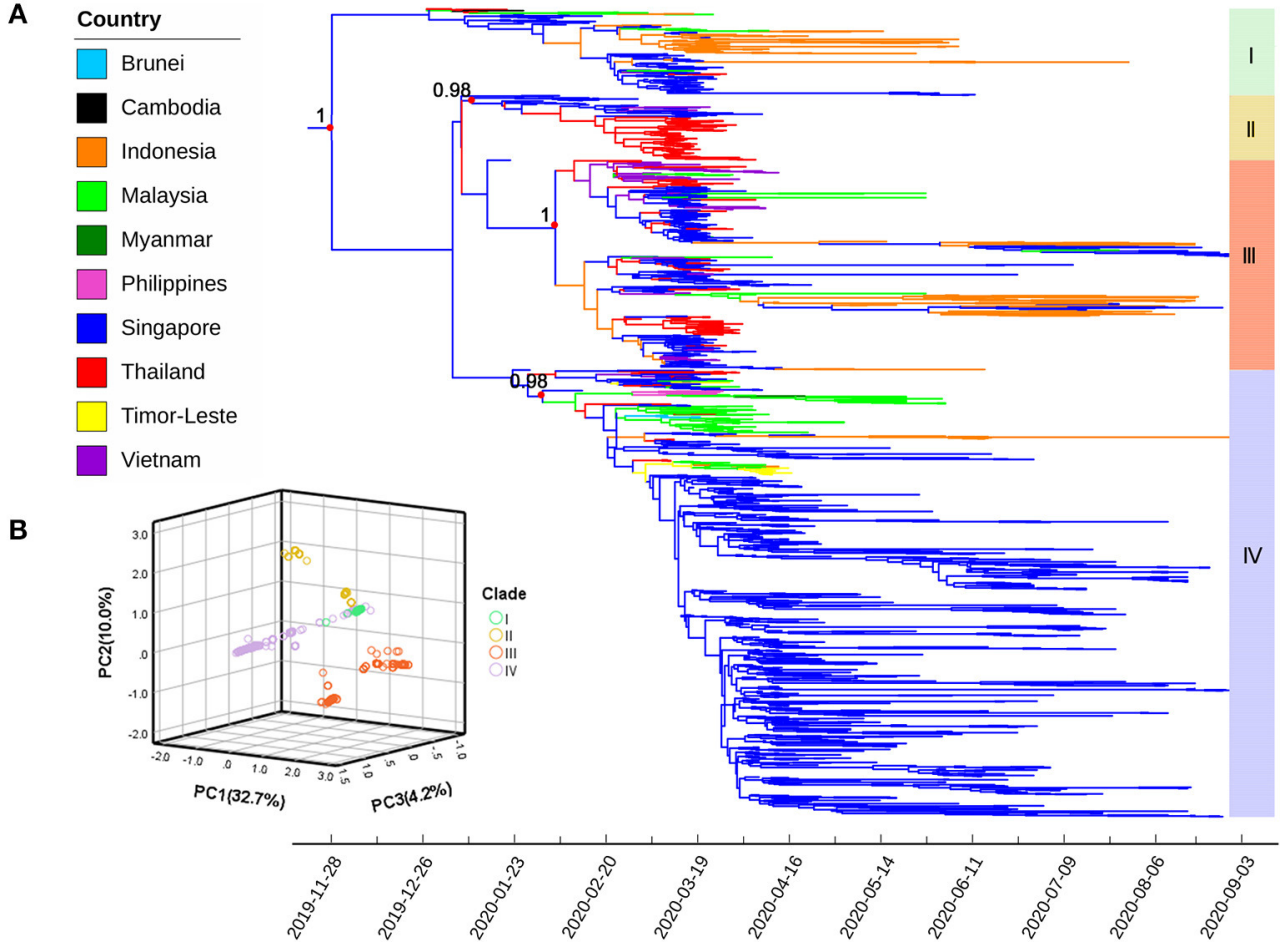
**(A)** Maximum clade credibility (MCC) tree of severe acute respiratory syndrome coronavirus 2 (SARS-CoV-2) strains isolated from Southeast Asia using complete genomes. Colors indicate different sampling locations. Red dots represent key nodes; the corresponding posterior probability values (> 0.95) are indicated. **(B)** The plot of the first three principal components (PCs) from the principal component analysis based on genomic variations; sample clustering is roughly in agreement with the MCC tree. Four major clades (I–IV) are marked with the same color scheme in both figures.

Phylogenetic relationships among 1,491 SARS-CoV-2 genomes were reconstructed using Bayesian methods and visualized in the form of an MCC tree (Figure 2A). The estimated root position and age were Singapore/Thailand (posterior probabilities 0.549 and 0.413, respectively) and November 28, 2019 [95% highest posterior density (HPD) interval: September 7, 2019 to January 4, 2020], respectively. Posterior probabilities for all Southeast Asian countries, as root positions, are presented in Supplementary Table 2. Furthermore, the evolutionary rate of SARS-CoV-2 isolated from Southeast Asia was estimated to be 1.446 × 10^−3^ (95% HPD: 1.292 × 10^−3^ to 1.613 × 10^−3^) substitutions per site per year.

Four distinct phylogenetic clades (labeled I–IV) were identified, which were consistent with the topological structure of the ML phylogenetic tree. Clades I and II were relatively early in terms of divergence time, containing fewer sequences, with 160 and 119 sequences for Clades I and II, respectively. Clade III had 387 sequences and presented high genetic diversity with two separate subclades formed in mid-February 2020 and had the widest distribution across Southeast Asia. Of the sequences, 55.3% clustered in Clade IV (number of sequences: 825), most of which originated from Singapore, followed by Malaysia. All sequences from this region were relatively concentrated in one or two clades, except those originating from Singapore.

The first three PCs explained 46.9% of the total genomic variations among the samples. A three-dimensional scatter plot of PC1 (32.7%), PC2 (10.0%), and PC3 (4.2%) revealed a clustering pattern that was roughly the same as the branching of the MCC tree (Figure 2B).

### Bayesian Phylogeographical Inference of SARS-CoV-2 in Southeast Asia

**Figure 3:**
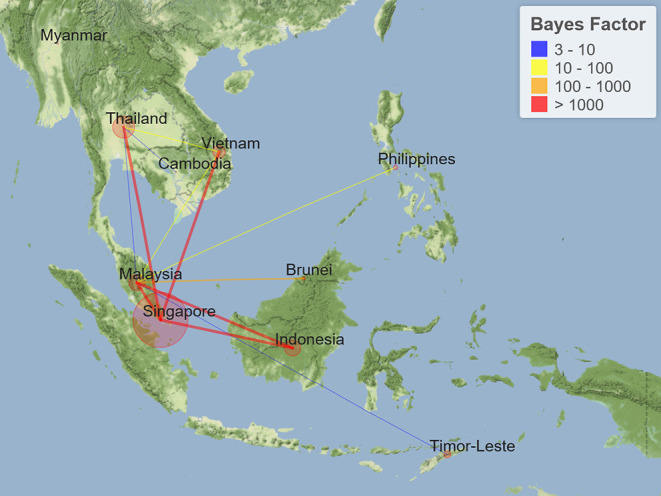
Transmission links of coronavirus disease 2019 (COVID-19) within Southeast Asia tested by Bayes factor (BF). Line colors represent the relative strength by which the rates are supported: very strong (BF > 1,000, red lines), strong (100 < BF < 1,000, orange lines), moderate (10 < BF < 100, yellow lines), and positive (3 < BF < 10, blue lines).

Probabilities of epidemiological associations among different sampling locations were calculated using the two-way asymmetric BSSVS; results are shown in Figure 3. During the study period, Singapore was estimated to have significant epidemiological associations with Thailand, Indonesia, Vietnam, and Malaysia, with BF values exceeding 1,000. In addition to neighboring Singapore, Malaysia was significantly associated with Indonesia, Brunei, the Philippines, Vietnam, Timor-Leste, and Thailand, with BF values ranging from 1,732 to 5. These associations make Malaysia the country with the highest number of transmission links in Southeast Asia. In mainland Southeast Asia (except Peninsular Malaysia), there were two estimated transmission links in the early stage: Thailand–Vietnam and Thailand–Cambodia, with BF values of 15 and 9, respectively. Significant transmission links with corresponding BF values are listed in Table 1. Additionally, time-varying transmission links were visualized on a dynamic map (http://gos.mingleadgene.com/gosweb/virusdb/dataChartBk.html).

**Table 1:** Two-way Bayes factor tests for SARS-CoV-2 transmission links in Southeast Asia.

**Transmission link**	Bayes factor^*^
Brunei-Malaysia	405
Cambodia-Thailand	9
Indonesia-Malaysia	1732
Indonesia-Singapore	652305
Malaysia-Philippines	14
Malaysia-Singapore	1088
Malaysia-Thailand	5
Malaysia-Timor-Leste	7
Malaysia-Vietnam	29
Singapore-Thailand	INFINITE
Singapore-Vietnam	203843
Thailand-Vietnam	15

* *Only significant non-zero transmission links (Bayes factor > 3) are listed*.

### Population Dynamics of SARS-CoV-2 in Southeast Asia

**Figure 4:**
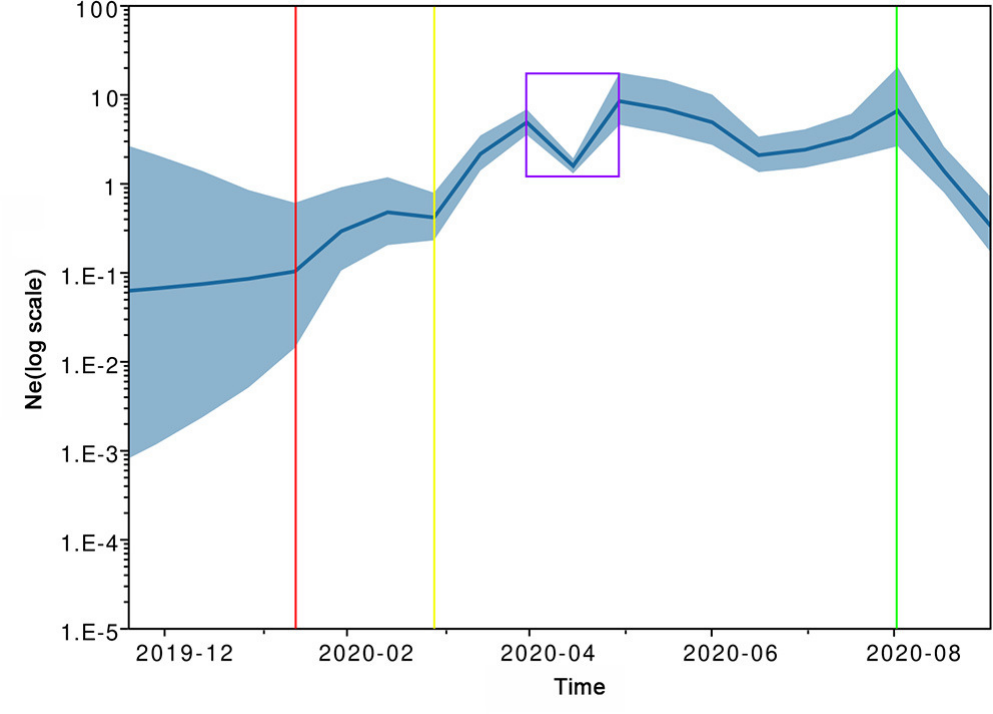
Historical changes in effective population size (Ne) of severe acute respiratory syndrome coronavirus 2 (SARS-CoV-2) circulating in Southeast Asia by September 2020. The blue line represents posterior point estimates; the blue band represents 95% Bayesian credible interval (BCI). The red, yellow, and green vertical lines mark the watersheds corresponding to two accelerated expansions and one significant contraction; the purple box marks a temporary setback followed by a rapid rebound.

The inferred effective population size trajectory of SARS-CoV-2 circulating in Southeast Asia is shown in the Bayesian Skygrid plot (Figure 4). From the tMRCA to late March 2020, the effective population of the virus generally grew in a continuous manner and with two accelerations (around mid-January 2020 and late February 2020, respectively). In April 2020, the population size underwent a sudden fluctuation and hit a lower point in the middle of that month. Over the following 3 months, the viral population seemed to remain relatively steady. A pronounced population contraction took place in August 2020.

## Discussion

To the best of our knowledge, this is the first study to investigate the molecular phylogeny and spatiotemporal spread of SARS-CoV-2 in Southeast Asia using the Bayesian framework. Phylogenetic relationships among 1,491 SARS-CoV-2 isolates from 10 Southeast Asian countries were determined by analyzing their complete genomic sequences. Furthermore, the regional transmission dynamics and effective population size changes during the first 8 months of 2020 were illustrated.

The previous finding that the phylodynamic threshold of SARS-CoV-2 was attained by February 2, 2020, provided a reasonable basis for us to infer its evolutionary history through genomic analyses (37). Although positive temporal signals had been detected in viral genome sequence data collected in the early stages, they were generally minor, with their corresponding R^2^ (in root-to-tip regressions) values being smaller than 0.2 (37, 38). The improved temporal structure of our dataset may be the consequence of sufficient accumulation of variations over a longer time span rather than an increase in the number of genome samples. Exact sampling dates were used, based on this feature, to calibrate the molecular clock.

Both Singapore and Thailand presented the highest posterior probabilities as root positions, with a slight gap and a sum exceeding 0.95. The first confirmed cases of COVID-19 in Southeast Asia were found in Thailand (January 13, 2020) and Singapore (January 23, 2020) (39). More importantly, these two countries were among the first within the region where local transmission of the virus emerged (on January 31, 2020 and February 3, 2020, respectively). There was no direct correlation between relevant infection clusters (40, 41). Thus, we speculate that the outbreak in this region probably originated in both places almost simultaneously, constituting a “dual origin” pattern. In the Western Hemisphere, Europe is another possible example of a within-region COVID-19 outbreak of dual origin. Evidence based on genomic epidemiology indicated that Italy and Germany might have been introduced to SARS-CoV-2 early and independently and seeded the epidemic in Europe (20, 42, 43). Bayesian time-scaled phylogenetic analysis with the tip-dating method revealed that rootage or the time to the most recent common ancestor (tMRCA) converged to late November 2019, which is consistent with reported estimates based on analyses of sequences collected worldwide (37, 38). However, tMRCA estimates varied regionally, as follows: mid-November 2019 for East Asia (18), early December 2019 for Africa (44), late December 2019 for Oceania and North America (20, 45), late January 2020 for Europe (20), and mid-February 2020 for South America (46). Such regional differences reflect the non-synchronicity of viral introductions globally. In comparison, our estimate for Southeast Asia seems to be the earliest (except for East Asia), despite its relatively broad 95% HPD interval. Notably, SARS-CoV-2 related coronaviruses (SC2r-CoVs), which share high nucleotide identity with SARS-CoV-2, have been detected in *Rhinolophus* bats from Southeast Asia. Thus, Southeast Asia could be an important candidate for the origin of SARS-CoV-2 (47, 48). The median evolutionary rate for SARS-CoV-2 isolates from Southeast Asia during the study was 1.446 × 10^−3^ substitutions per site per year, which is slightly higher than the results (~1.1 × 10^−3^) reported in most studies (37, 38, 45, 49, 50). It is unclear whether this subtle difference is related to lineage composition or climatic conditions. However, it was significantly higher than that reported for early African strains, with a median of 4.133 × 10^−4^ substitutions per site per year (44). This difference seems to be largely attributable to the estimation bias caused by considering only early viral sequences with a small sample size in the African study.

The evolutionary branches in the phylogenetic tree are a manifestation of genetic diversity. However, determining distinct clades or subgroups is a major challenge in analyzing genetic datasets (51). In this study, the MCC tree showed that SARS-CoV-2 strains circulating in Southeast Asia had evolved into at least four major clades, a finding supported by PCA results and sample points labeled with the major clades of the MCC tree clustered in the corresponding PCA plot. Notably, the first PC (PC1) from PCA explained nearly one-third of total genomic variation among samples, indicating limited genetic diversity in SARS-CoV-2 isolates from Southeast Asia. This finding was consistent with a global-level study and could be interpreted as the effect of pure genetic drift (52). Most of the analyzed samples were from Singapore and were incorporated into all clades. Singapore, an international financial center and free trade port, has a well-developed transportation network. Many studies have highlighted the role of travel (especially international air travel) in promoting the spatial transmission of SARS-CoV-2 (53, 54), which can be used to explain the diverse lineage composition of virus strains in Singapore. Notably, we found that the largest clade, namely Clade IV, appeared to have a continuous and relatively independent evolution centered in Singapore, despite its late divergence time. According to GISAID, up to 96.0% of sequences within Clade IV belong to GISAID-Clade O (O meaning “Others”), whereas 3.6% belong to GISAID-Clade V (55). Therefore, this clade likely represents a unique group of variants evolved from GISAID-Clade V and warrants further analysis. Most strains from Thailand were clustered in Clade II, which was evolutionarily older and roughly corresponded to GISAID-Clade S under the framework of GISAID. In the early days of the epidemic, Chinese scientists reported that GISAID-Clade S strains resulted from relatively conservative evolution and presented low infection rates (56). This is a potential explanation for why COVID-19 in Thailand was more effectively contained than in other countries (11, 40). A high concentration in one subclade of Clade III was observed for the strains from Vietnam, and all belonged to GISAID-Clade GR. GISAID-Clade GR, with the genetic marker S-D614G, was dominant in the WHO European Region (57), and the second wave of COVID-19 in Vietnam was triggered by a series of cases imported from Europe during March 2020, when approximately 85% of Vietnamese strains included in this study were sampled (58). The results of our study add circumstantial evidence to such a transmission link between Vietnam and Europe. Indonesia, the most populous country in Southeast Asia, is the world's largest island country and comprises more than 17,000 islands. Phylogenetic analysis revealed that its lineage composition was also very diverse, with the coexistence of both old and new strains, even if they were relatively concentrated in only two evolutionary clades (Clade I and a subclade of Clade III). This could be the result of spatially and temporally separate introduction events related to fragmented topography (59).

Quantitative and visualizing transmission links were further inferred by Bayesian phylogeographical analysis based on molecular associations among SARS-CoV-2 isolates from different countries in Southeast Asia. In maritime Southeast Asia, the high-support transmission links with BF values exceeding 1,000 in Singapore, Malaysia, and Indonesia have constituted a “transmission triangle” since March 2020. This epidemic pattern could be supported by the surveillance results of the Ministry of Health (MOH) Malaysia. As of August 27, 2020, the first and third countries of origin of imported COVID-19 cases in Malaysia were Indonesia and Singapore, respectively (60). Interestingly, a triangular cross-border regional development plan (the Indonesia–Malaysia–Singapore Growth Triangle, IMS-GT) has been in effect for more than 20 years, strengthening the integration of capital, land, and labor between these countries. Such a pattern of cross-border transmission is more likely to be attributable to certain negative externalities of the development of IMS-GT than simply geographical proximity (61). Sri Petaling tabligh, a large religious gathering, took place in Malaysia at the end of February 2020, which subsequently triggered the widespread of COVID-19 in Southeast Asia (62). It was reported that the 4-month-long infection cluster eventually led to 2,550 Malaysian cases from 7 states and 825 non-Malaysian cases from 28 countries (63). At the molecular level, results from our phylogeographical analysis suggested that Malaysia possessed transmission links with seven other countries in the region, which is consistent with the colocalization of related isolates in the MCC tree. By combining macro- and micro-epidemiological evidence, Malaysia was inferred to be a potential regional epicenter during the early stages of the COVID-19 outbreak. In mainland Southeast Asia, two early transmission links in Thailand, Vietnam, and Cambodia were probably indirect, considering that most of their early cases were imported from China (40, 58, 64). Both Bayesian phylogenetic analysis and BF analysis revealed that the Thai strains were closely related to the Singaporean strains. Singapore is one of the largest destination countries for labor export from Thailand (65). According to the Royal Thai Ministry of Public Health (MOPH), some Thai nationals working in Singapore returned during the pandemic and subsequently tested positive for SARS-CoV-2 (66). Hereby, we speculated that the mobility of migrant workers could contribute in part to the Singapore–Thailand transmission link (67).

The effective population size represents the number of diverse genomes capable of producing progeny virions and indirectly reflects the number of infected individuals in the context of the epidemic (68, 69). The Bayesian Skygrid model is an improved coalescent-based model with higher accuracy in recovering population size trajectories than Bayesian Skyline and Skyride models (70). Using the Bayesian Skygrid model, we found that the SARS-CoV-2 circulating in Southeast Asia experienced a complex demographic history. In line with the recent origin of the virus, the initial effective population size of Southeast Asian SARS-CoV-2 was low (71). After a potential “adaptation period” of cross-species transmission (72), the virus experienced its first accelerated population expansion in mid-January 2020, when the first infection in Southeast Asia was confirmed (39). The second accelerated expansion of the viral population occurred in March 2020, matching the exponential growth pattern of reported cases during the same period (66). Epidemiological investigations confirmed that the early representative infection clusters in this region involved multiple mass gatherings (MGs) or superspreading events (SSEs), such as the SAFRA Jurong cluster in Singapore (involving a private dinner function held on February 15, 2020), Sri Petaling Tabligh cluster in Malaysia (involving an Islamic missionary convention held from February 27 to March 3, 2020) and Lumpinee Boxing Stadium cluster in Thailand (involving a Thai boxing match held on March 6, 2020) (8, 9, 40). These MG/SSE-related clusters were likely to be important external drivers of viral exponential expansion (73–75). It took Southeast Asian countries an average of 17 days to declare a state of emergency or lockdown after 50 cases were confirmed (76). Our reconstruction also showed that the population expansion of Southeast Asian SARS-CoV-2 suffered a temporary setback in April 2020, and the corresponding 95% Bayesian credible interval was narrow. Accordingly, this phenomenon may reflect the efficacy of public health interventions (e.g., Movement Control Order of Malaysia effective from March 18, 2020, and the nationwide isolation order of Vietnam effective from April 1, 2020) (58, 63). However, the rapid rebound in effective population size not only highlighted the impact of shifting transmission dynamics and hidden reservoirs (68) but also underscored the importance of integrated and sustainable response strategies (53). The final population contraction, which was unexpected, possibly arose from bias due to the small sample size (18, 44).

This study has two basic limitations. First, the relatively small and unevenly distributed sample complicated the inference for the spatiotemporal patterns of viral spread in the region, as observed in all molecular epidemiological studies using real-world data (18). For example, only seven sequences were available from the Philippines, one of the worst-affected Southeast Asian nations, meaning more potential transmission links involving the Philippines remain unresolved in our model (9). We only selected the first 8 months of the epidemic to minimize sampling biases, as the research period based on the tradeoff between sampling coverage and sampling density (77, 78). Except for a few excluded sequences due to data quality control, we included nearly all the Southeast Asian SARS-CoV-2 sequences available then. Second, the virus importations from other regions may pose a potential threat to the validity of the research findings. As the COVID-19 pandemic continues to spread worldwide, it is unlikely that the SARS-CoV-2 strains circulating in any region are entirely indigenous. Unfortunately, we failed to adjust for this confounding factor directly because travel history was only available for four sequences in the dataset. However, the impact of the interregional importations of the virus seems to be relatively limited. Empirical evidence shows that compared with the long-distance spread across regions, SARS-CoV-2 is much easier to spread between neighboring countries over short distances (22, 79, 80). For Southeast Asia, international travel bans were announced at a very early stage of the pandemic (76, 81), but movement restrictions within the region, the focus of this research, were relatively loose (81). Indeed, genomic epidemiology is an important complement to traditional epidemiological investigations rather than a substitute (13). Therefore, we combined the results with macro-epidemiological statistics to a large extent and adopted a cautious approach throughout the inference process. Further investigations into the relationship between the molecular evolution of SARS-CoV-2 and its spread patterns are warranted using a more representative sample and in a broader context.

## Conclusions

In conclusion, this study provides insights into the genomic epidemiology of SARS-CoV-2 in Southeast Asia, a tropical region with limited resources. The most recent common ancestor of Southeast Asian strains was estimated to have emerged in Singapore and/or Thailand since late November 2019. Our phylogenetic analysis revealed an average evolutionary rate of 1.446 × 10^−3^ substitutions per site per year and at least four major evolutionary branches (one of which was potentially endemic). Moreover, the spatial spread of SARS-CoV-2 within this region was characterized by a potential epicenter (Malaysia) and an inferred “transmission triangle” (Indonesia–Malaysia–Singapore). The population dynamics during the first 8 months presented a fluctuating process with early expansion. These findings have significant implications for public health interventions. Continuous-genomic surveillance and enhanced strategic collaboration should be listed as priorities in combatting the ongoing pandemic, especially for regional communities dominated by developing countries.

## Data Availability Statement

The original contributions presented in the study are included in the article/Supplementary Material, further inquiries can be directed to the corresponding author/s.

## Author Contributions

LL, MZ, JS, and QZ designed the study. JK and IC downloaded and aligned the sequence data. MZ, JS, JL, and NL constructed the evolutionary trees. QZ, JT, JK, and AT performed the phylogeographical analysis. QZ, IC, and SC estimated the population size. JT and JK visualized the results. SC and AT independently checked the validation of all calculations. MZ, JS, and IC managed the literature searches and wrote the original draft. LL and JT reviewed and modified the manuscript. All authors contributed to and have approved the published version of the manuscript.

## Conflict of Interest

The authors declare that the research was conducted in the absence of any commercial or financial relationships that could be construed as a potential conflict of interest.

## Publisher's Note

All claims expressed in this article are solely those of the authors and do not necessarily represent those of their affiliated organizations, or those of the publisher, the editors and the reviewers. Any product that may be evaluated in this article, or claim that may be made by its manufacturer, is not guaranteed or endorsed by the publisher.

## Supplementary Material

Supplementary Figure 1 Workflow schema describing key implementation steps and the logic. Steps connected by solid lines constitute the main technical route of the study, whereas those connected by dotted lines represent auxiliary or verification steps.

Supplementary Table 1 Distributions of the SARS-CoV-2 sequence samples included in this study.

Supplementary Table 2 Root probability and outbreak date for each country in Southeast Asia.
