# Supplementary figures and images for: Molecular Phylogenesis and Spatiotemporal Spread of SARS-CoV-2 in Southeast Asia
Source: Front Public Health. 2021 Jul 30;9:685315. doi: 10.3389/fpubh.2021.685315 (PMC8363229; doi:10.3389/fpubh.2021.685315)

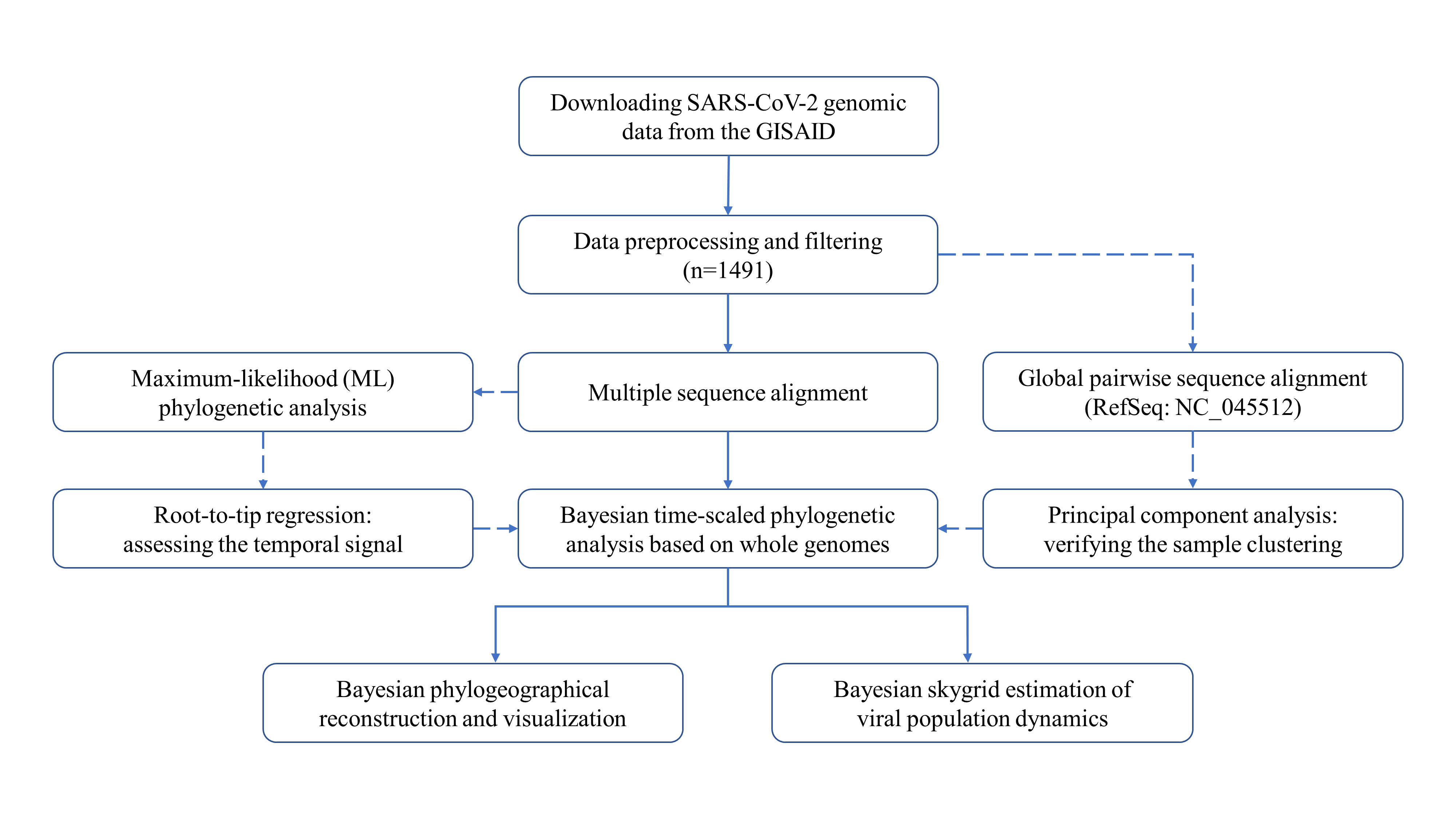

Supplement: Supplementary Figure 1 — Workflow schema describing key implementation steps and the logic. Steps connected by solid lines constitute the main technical route of the study, whereas those connected by dotted lines represent auxiliary or verification steps. [file Image_1.TIF]
