# Supplementary material for: Molecular Phylogenesis and Spatiotemporal Spread of SARS-CoV-2 in Southeast Asia
Source: Front Public Health. 2021 Jul 30;9:685315. doi: 10.3389/fpubh.2021.685315 (PMC8363229; doi:10.3389/fpubh.2021.685315)
Supplement: Supplementary Table 1 — Distributions of the SARS-CoV-2 sequence samples included in this study. [file Table_1.DOCX]

**Table S1.** Distributions of the SARS-CoV-2 sequence samples included in this study.

| Distribution by country | | Distribution by month/2020 | |
| --- | --- | --- | --- |
| Singapore | 1058 | January | 16 |
| Thailand | 169 | February | 34 |
| Malaysia | 95 | March | 613 |
| Indonesia | 89 | April | 426 |
| Vietnam | 52 | May | 106 |
| Timor-Leste | 14 | June | 116 |
| Others* | 14 | July | 112 |
|  |  | August | 68 |
| Total | 1491 | | |

* The remaining samples include seven samples from the Philippines, five from Brunei and one from Cambodia and Myanmar.
