# Supplementary material for: Molecular Phylogenesis and Spatiotemporal Spread of SARS-CoV-2 in Southeast Asia
Source: Front Public Health. 2021 Jul 30;9:685315. doi: 10.3389/fpubh.2021.685315 (PMC8363229; doi:10.3389/fpubh.2021.685315)
Supplement: Supplementary Table 2 — Root probability and outbreak date for each country in Southeast Asia. [file Table_2.DOCX]

**Table S2.** Root probability and outbreak date for each country in Southeast Asia.

| Country | Posterior probability (as root) | Date of the1st case * |
| --- | --- | --- |
| Brunei | 0.0002 | 9 March 2020 |
| Cambodia | 0.0020 | 27 January 2020 |
| Indonesia | 0.0014 | 2 March 2020 |
| Malaysia | 0.0139 | 25 January 2020 |
| Myanmar | 0.0000 | 23 March 2020 |
| Philippines | 0.0002 | 30 January 2020 |
| Singapore | 0.5490 | 23 January 2020 |
| Thailand | 0.4132 | 13 January 2020 |
| Timor-Leste | 0.0003 | 21 March 2020 |
| Vietnam | 0.0198 | 23 January 2020 |

* Source: ASEAN Biodiaspora Virtual Center (ABVC). Risk Assessment for International Dissemination of COVID-19 to the ASEAN Region. https://asean.org/storage/2020/02/COVID-19-Risk-Assessment_ASEAN-BioDiaspora-Regional-Virtual-Center_10Apr2020.pdf.
